# Specific and Plastic: Chandelier Cell-to-Axon Initial Segment Connections in Shaping Functional Cortical Network

**DOI:** 10.1007/s12264-024-01266-3

**Published:** 2024-07-30

**Authors:** Yanqing Qi, Rui Zhao, Jifeng Tian, Jiangteng Lu, Miao He, Yilin Tai

**Affiliations:** 1 Institutes of Brain Science, State Key Laboratory of Medical Neurobiology and MOE Frontiers Center for Brain Science, Department of Neurobiology, Zhongshan Hospital, Fudan University, Shanghai, 200032 China; 2 Songjiang Research Institute, Shanghai Songjiang District Central Hospital, School of Medicine, Shanghai Jiao Tong University, Shanghai, 200025 China; 3 Center for Brain Science of Shanghai Children’s Medical Center, Department of Anatomy and Physiology, School of Medicine, Shanghai Jiao Tong University, Shanghai, 200127 China

**Keywords:** Axon initial segment, Chandelier cell, Plasticity

## Abstract

Axon initial segment (AIS) is the most excitable subcellular domain of a neuron for action potential initiation. AISs of cortical projection neurons (PNs) receive GABAergic synaptic inputs primarily from chandelier cells (ChCs), which are believed to regulate action potential generation and modulate neuronal excitability. As individual ChCs often innervate hundreds of PNs, they may alter the activity of PN ensembles and even impact the entire neural network. During postnatal development or in response to changes in network activity, the AISs and axo-axonic synapses undergo dynamic structural and functional changes that underlie the wiring, refinement, and adaptation of cortical microcircuits. Here we briefly introduce the history of ChCs and review recent research advances employing modern genetic and molecular tools. Special attention will be attributed to the plasticity of the AIS and the ChC-PN connections, which play a pivotal role in shaping the dynamic network under both physiological and pathological conditions.

## Introduction

Within a neuron, the axon initial segment (AIS) stands out as the most excitable subcellular compartment where action potentials are readily initiated [1–5]. Therefore, modulating AIS is an efficient way to adjust neuronal output and excitability. Indeed, neurons have adopted sophisticated ways to modulate the morphology and position of AIS, as well as the composition of ion channels within the AIS, in response to changes in network activity [6–8]. These adaptive changes shape the kinetics of action potentials and fine-tune neuronal excitability to maintain network stability or to reshape the strength of specific synaptic connections. Therefore, the plasticity of AIS is a critical form of neuronal plasticity on the subcellular level that contributes to the overall flexibility of the neural network in response to internal or external stimuli.

**Fig. 1:**
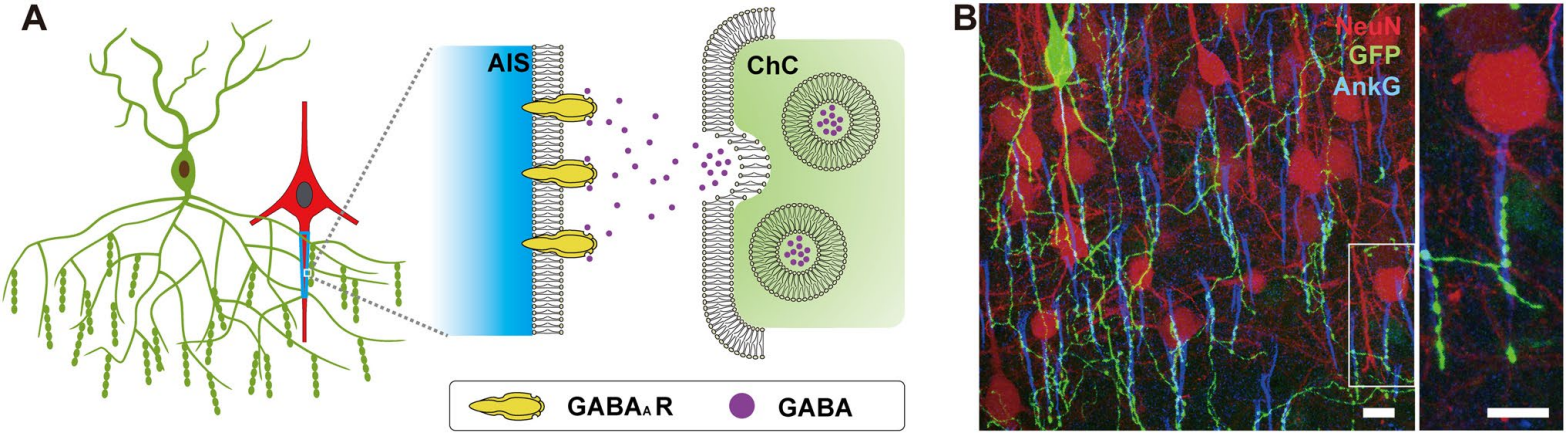
The morphological features and targeting selectivity of chandelier cells.** A** Schematic of a chandelier cell (ChC) (green cell) innervating axon initial segments (AISs) (aqua trapezium) of nearby projection neurons (PNs) (red cell), forming the characteristic axonal cartridges composed of strings of presynaptic boutons. The right schematic diagram is the ultrastructure of axo-axonic synapse, showing the postsynaptic (AIS), synaptic cleft, and presynaptic (cartridge), from left to right. **B** Representative image of a layer 2 ChC in the somatosensory cortex. The ChC was labeled by GFP (green channel), the nearby PNs were labeled by TdTomato (red channel), and the AISs were labeled by AnkG staining (blue channel). The zoomed-in image displays a PN’s AIS innervated by a ChC cartridge. Scale bar: 10 μm.

**Fig. 2:**
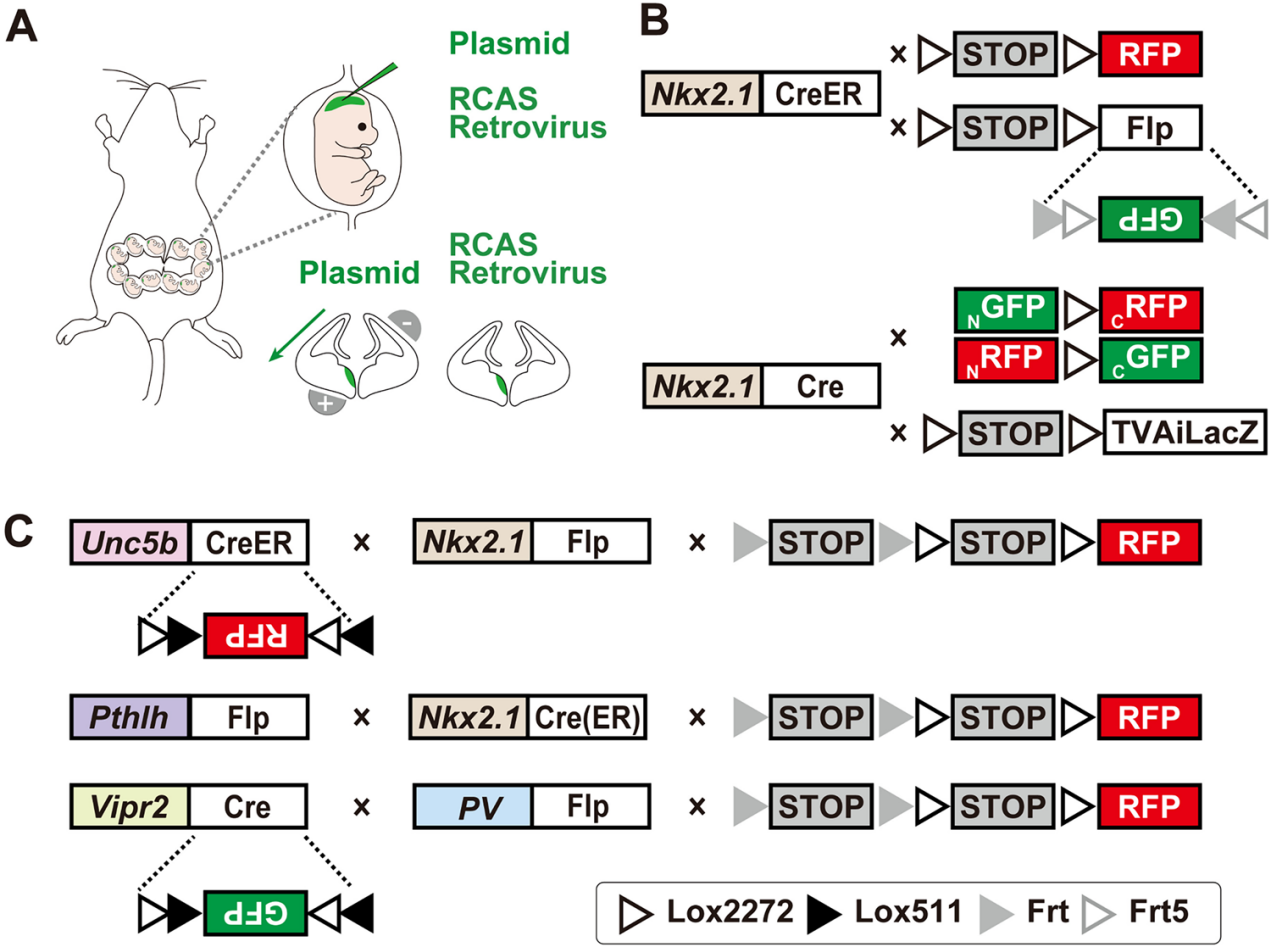
Non-genetic and genetic methodologies to label chandelier cells. **A** ChCs are labeled by in utero plasmid transfection or viral injection into progenitors residing in the medial ganglionic eminence (MGE). **B** ChCs are labeled by genetic fate mapping of MGE progenitors expressing the transcriptional factor Nkx2.1. Late embryonic induction of Nkx2.1-CreER labels ChCs in the neocortex with relatively high specificity. Combining it with the Ai14 reporter enables fluorescent labeling and direct visualization while combining it with a LoxP-STOP-LoxP-Flp reporter converts the transient CreER expression into constitutive Flp expression to activate Flp-dependent gene expression in AAV-infected ChCs. Nkx2.1-Cre is not specific to ChCs, but combining it with MADM enables clonal analysis of MGE progenitors. ChCs are the final output from the last neurogenic divisions and can be identified by their unique morphology. Alternatively, ChCs can be labeled by combining Nkx2.1-Cre with retrovirus injection during the late embryonic stage, along with the cre-dependent TVAiLacZ line. **C** ChCs are labeled by drivers targeting marker genes identified by single-cell transcriptomic studies: Unc5b-CreER, Pthlh-Flp, and Vipr2-Cre. Intersection with orthogonal drivers, or using viral reporters instead of mouse reporters, further improved the labeling specificity.

**Fig. 3:**
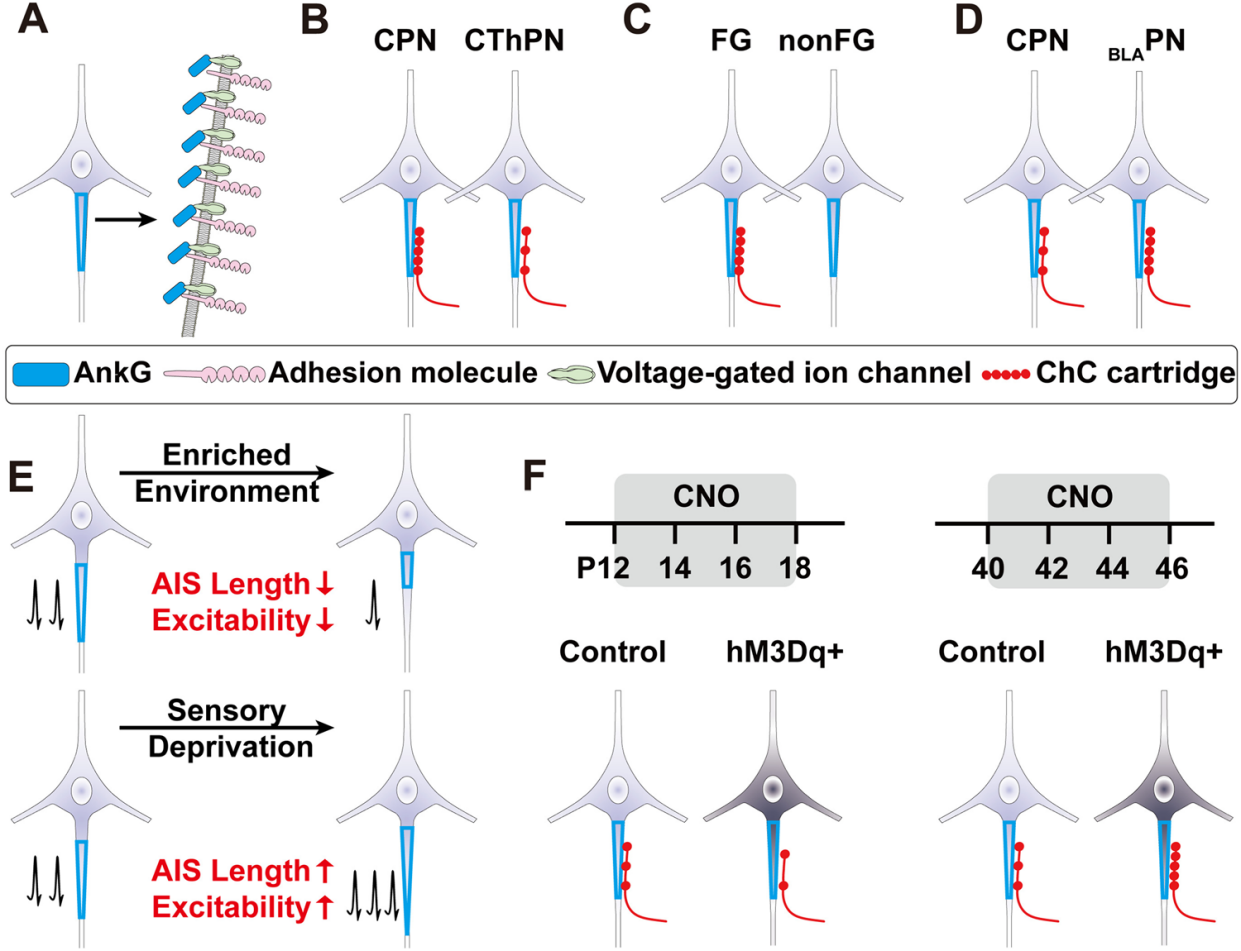
Plasticity and Specificity of the ChC-AIS connections. **A** Schematics illustrating the structure and composition of the AIS. AnkG is the master organizer which acts as an anchor protein to recruit and organize adhesion molecules and voltage-gated ion channels for action potential generation and propagation. **B** In the visual and auditory cortices of cats, callosal projection neurons (CPNs) exhibit a higher synaptic density on their AIS compared to corticothalamic projection neurons (CThPNs). **C** In the piriform cortex of mice, the AIS of centrifugal cells (FGs) that project to the main olfactory bulb display a higher number of synapses compared to non-projecting non-centrifugal cells (nonFGs). **D** In the prelimbic cortex of mice, ChCs preferentially innervate the basolateral amygdala-projecting neurons (BLAPNs) compared to the contralateral prelimbic cortex-projecting neurons (CPNs). **E** Homeostatic plasticity of the AIS in response to network activity changes. When network activity increases, the AIS shortens and neuronal excitability decreases. Conversely, when network activity decreases, the AIS elongates and neuronal excitability increases. **F** Homeostatic plasticity of the axo-axonic synapses at the AIS during early developmental stage and adulthood: Persistent chemogenetic activation of PNs from P12 to P18 leads to decreased innervation of ChCs at the AIS, whereas prolonged activation of PNs from P40 to P46 results in increased ChC-AIS innervation.

In the cerebral cortex, hippocampus, and basolateral amygdala (BLA) of mammals, the AISs of PNs receive innervation from a distinct type of GABAergic interneurons called chandelier cells (ChCs), also known as axo-axonic cells (AACs) (Fig. 1) [9–12]. This unique and specialized connection is believed to play a crucial role in regulating neuronal output. However, progress in understanding the development, connectivity, and function of ChCs has been limited since their discovery in 1974, primarily due to the lack of tools for precise labeling and manipulation. Recent advancements in genetic labeling of ChCs have provided new opportunities for significant breakthroughs. These techniques are based on either developmental origin revealed by genetic fate-mapping or molecular markers identified through single-cell RNA sequencing (Fig. 2) [13–28]. Studies utilizing these techniques have provided insights into the developmental trajectory, molecular and cellular characteristics, and anatomical and functional connectivity of ChCs. Moreover, they begin to uncover plastic changes in the exclusive ChC-to-AIS connections, in response to and influences on changing local and network activity in both healthy and diseased brains (Fig. 3) [16, 29–33].

Here, we will first introduce a brief history of ChCs and their basic features. We will then summarize the traditional and newly-developed methodologies employed to study ChCs, along with an outline of the current understanding of the cellular and molecular mechanisms underlying the specificity of ChC-AIS connectivity. Subsequently, we will delve into recent studies that have investigated the plasticity of AISs, ChCs, and the ChC-AIS connections in the developing and mature neural circuits under physiological and pathological conditions.

## A Brief History and the Basic Features of ChCs

As a numerical minority within the GABAergic interneuron family, ChCs were discovered much later than other cortical interneuron classes. In the 19th century, Cajal and Lorente may have encountered immature ChCs when dissecting young mammalian brains but classified them as atypical basket cells [34]. It was not until the 1970s that Szentágothai and Arbib discovered ChCs in the cingulate gyrus cortex of cats and named them "chandelier cells" due to their resemblance to Western-style chandeliers [12, 35]. At almost the same time, Jones also discovered in the cortex of squirrel monkeys the presence of type 4 cells with axon terminals that are vertically oriented and exhibit bead-like structures [11]. Initially, Szentágothai speculated that ChCs targeted the apical dendrites of PNs [35]. However, this view was later challenged by Somogyi *et al.*, who used electron microscopy to demonstrate that ChCs synapse on the AIS of PNs, leading to the alternative name “axo-axonic cells” [36]. Subsequent studies confirmed the presence of ChCs in cortical structures across various mammalian species, ranging from mice and rats to monkeys and humans, but not yet in non-mammalian species [36–44]. Therefore, ChCs are believed to be a mammalian-specific cell type that potentially plays an important role in higher-order brain functions.

A recent study has unveiled that ChCs are present throughout structures derived from the pallium in the mouse brain, including the cerebral cortex, hippocampal formation, claustrum-insular complex, extended amygdaloid complex, and olfactory centers [17]. Within the cortex, the distribution of ChCs shows distinct laminar bias and regional variations. The majority of ChCs reside right below the L1/2 boarder and innervate local PNs in the superficial layers, but some ChCs extend their axon arbors into deeper layers of the cortex. Deep cortical layers have a much sparser ChC distribution. Ventrolateral cortical regions, including the piriform cortex, anterior insula cortex, entorhinal cortex, perirhinal cortex, and temporal association cortex, as well as the infra-limbic area of the mouse prefrontal cortex, exhibit higher ChC density compared to other cortical regions [17].

The most distinctive morphological feature of ChCs is their axonal “cartridges”, which are strings of presynaptic boutons running along the AIS of targeted PNs. The AIS receives exclusively GABAergic synaptic input. Each cartridge typically consists of several to a dozen presynaptic boutons, forming classic symmetric synapses on the AIS (Fig. 1). ChCs have highly complex axon arborizations that allow them to innervate hundreds of PNs [42, 45]. Therefore, the axon morphology of ChCs closely mirrors the anatomical organization of the PNs they innervate. In brain regions with clear laminar organization, such as the cerebral cortex, ChCs exhibit the typical “chandelier lamp” morphology, with their axon cartridges arranged in a distinct pattern (Fig. 1). In regions lacking well-defined cellular layers, such as the BLA, the morphology of ChCs is less uniform and tends to be more multipolar in nature [46].

Although ChCs were initially identified based on their distinct morphology, subsequent discoveries revealed that ChCs express parvalbumin (PV), one of the markers for GABAergic subtypes. Together the fast-spiking feature of ChCs has led to their classification as a subgroup of PV-positive fast-spiking interneurons, which also includes PV basket cells. Recent genetic fate mapping and transcriptome analyses have challenged this classification. Labeling ChCs with genetic methods or performing transcriptomic clustering has revealed that a significant proportion of ChCs do not express detectable levels of PV. This raised questions about whether ChCs can be classified as a unique interneuron cell type and whether they can be further divided into additional subgroups. Since ChCs specifically innervate the AIS of PNs, while no other known interneuron type possesses this specificity, we can categorize ChCs as a distinctive interneuron type in this perspective. Nevertheless, it is important to acknowledge that subtypes of ChCs may exist, as they display morphologic heterogeneity that allows them to innervate different laminar regions, and a subgroup of them is capable of bilaminar innervation [47]. Single-cell spatial transcriptome analyses have revealed 3 subtypes of ChCs with preferential laminar locations and glutamatergic neurons being innervated in the macaque cortex. Among these subtypes, PV expression differs, with the lowest count in the subtype that is preferentially located in layer 2 [48]. Despite this, physiological and functional studies of molecularly defined ChC subtypes are not yet available. Considering the classification of ChC subgroups when studying the functions and projections of ChCs constitutes a critical future direction in this field.

## Methodologies to Study ChCs

### Non-genetic Approaches

For a few decades following the discovery of ChCs, researchers primarily relied on anatomical features to identify and study these cells. ChCs are often identified based on their stereotypical chandelier-like morphology or their exclusive AIS-targeting cartridges. In some studies, randomly labeled ChCs by Golgi staining or intracellular dye-loading were reconstructed from tissue blocks [49, 50]. In some other studies, ChCs were identified from datasets acquired by electron microscopy (EM) and were usually not fully reconstructed but partially traced back from AIS-targeting cartridges [25, 38]. These early studies provide insights into the distribution of ChCs or their axonal cartridges across different species and brain regions, as well as the structure properties of their AIS-specific synapses. More recently, advancements in high throughput three-dimensional (3D) EM imaging have enabled the mapping of postnatal development of cortical circuits [51]. These studies have provided a better understanding of the maturation of ChC-AIS synapses and their structural changes over time.

In human and non-human primate brain samples, a subset of GABAergic markers, such as PV and vesicular gamma-aminobutyric acid transporter (vGAT), allow axonal cartridges to be readily identified by immunostaining. The application of these markers in rodent brains is less straightforward and may not label the complete neuronal morphology. Nevertheless, this approach has been valuable in investigating pathological changes in cartridge density and morphology associated with brain disorders such as schizophrenia, epilepsy, autism spectrum disorder (ASD), and Alzheimer’s disease [32, 52–54]. ChCs were initially considered a subclass of PV cells due to their expression of PV and their shared embryonic developmental origin with other PV cells from the medial ganglionic eminence (MGE). As PV is mainly expressed in ChCs and PV-positive fast-spiking basket cells (PVBCs) but not in other interneuron classes, it can be used in combination with a second marker that distinguishes ChCs from PVBCs. For example, in the hippocampus, all PVBCs exhibit positive staining for SATB1, while ChCs were found to be SATB1 negative [55]. In the prefrontal cortex, vicia villosa lectin (VVA) has been used to detect the presence of N-acetylgalactosamine in the perineuronal net surrounding PVBCs but not ChCs [31]. Therefore, PV in combination with SATB1 immunostaining or VVA staining has been employed to label PVBCs or PV+ ChCs in these respective brain regions.

Leveraging the power of lineage tracing, ChCs have been labeled along with other interneuron subtypes by transfecting MGE neural progenitors through in-utero electroporation (IUE), infecting them through in utero retroviral injections, and by transplanting MGE into the postnatal cortex (Fig. 2A) [56–58]. These approaches have also enabled genetic manipulations of ChCs through shRNA-mediated gene knockdown, Cre recombinase-dependent gene knockout, or gene overexpression [57, 58]. Such studies have identified molecular candidates involved in the establishment and maintenance of AIS-specific synapses in ChCs [57, 58], which will be introduced in more detail in the next section. It is important to note that the success of these techniques relies heavily on the skills and experience of the investigator and their targeting efficiency and specificity for ChCs are quite limited. Similar to the Golgi staining and dye-loading methods, the identification of ChCs still relies on morphological analysis of the labeled cells.

There has been a lag in the investigation of the functional features of ChCs compared to studies on their anatomical characteristics. It was not until the 1990s that the electrophysiological properties of ChCs began to be reported. These studies involved blind recordings in acute hippocampal slices, combined with biocytin injection and *pos-hoc* morphological reconstructions [59, 60]. Electrophysiological studies revealed that ChCs exhibited fast-spiking firing properties similar to PVBCs, but with a more depolarized resting membrane potential and higher membrane resistance [19, 49]. Later, in vivo recordings shed light on the firing patterns of ChCs in relation to network oscillations and their responses to sensory stimuli in the cortex and hippocampus [50, 55, 61]. While these studies provided valuable insights into the intrinsic and functional features of mature ChCs, the recording efficiency was low, and technical demands were high, limiting in-depth systematic investigations. Similar to in vitro recordings in brain slices, ChCs were identified post-hoc based on their morphology. Since the morphological features of immature ChCs are less distinctive compared to mature ones, it is impossible to apply these methods to track the functional maturation of ChCs. Furthermore, these approaches are not suitable for long-term monitoring or activity modulation.

In summary, these non-genetic approaches share a common limitation: lack of ChC-targeting specificity. Therefore, the identification of ChCs relied on morphological features analyzed in post-modem samples. The lack of efficient and specific labeling methods that enable reliable identification, monitoring, and manipulation of ChCs, particularly in long-term and in vivo studies, has significantly impeded our understanding of their development and function.

### Genetic Approaches

In recent years, genetic strategies utilizing endogenous gene expression regulatory elements to drive transgene expression have been successfully employed to target various neuronal cell classes [20]. These strategies typically target either transcription factors that determine or are associated with specific neuronal identities, or marker genes that are differentially expressed in postmitotic cell types [21, 62]. A significant breakthrough in the genetic targeting of ChCs originated from a fate-mapping study that unexpectedly labeled ChCs with high specificity and efficiency using late embryonic induction of Nkx2.1-CreER, a driver line that targets the MGE-specific transcriptional factor Nkx2.1 (Fig. 2B) [13]. This discovery unveiled the presence of a progenitor domain in the late-stage embryonic ventral forebrain called the ventral germinal zone (VGZ), which predominantly generates ChCs. While this method does not exclusively label ChCs, it exhibits remarkably high labeling specificity in the cortex, particularly in the medial prefrontal region (above 90%). Consequently, it enables longitudinal tracking of ChC production, migration, apoptosis, and maturation, while also providing crucial experimental access for molecular and functional investigations.

Since Nkx2.1 expression is restricted to neural stem cells (NSC), subsequent studies have converted the NSC-specific CreER expression into constitutive Flp (Fig. 2B) [22]. Such conversion greatly expands the utility of this genetic labeling method, allowing for post-mitotic manipulations in ChCs using Flp viruses carrying various tools for anatomical or functional studies (Fig. 2B). Indeed, this approach has been used to analyze the morphological diversity of ChCs and trace the local and long-range presynaptic inputs of ChCs in the prefrontal cortex [16, 19, 23].

Despite its lack of specificity, Nkx2.1-Cre has also been employed to investigate the development of ChCs. It has been combined with MADM (mosaic analysis with double markers) reporter or retroviral reporter with low recombination efficiency to achieve sparse labeling of radial glia cells and lineage tracing of their progenies (Fig. 2B) [18]. Woodruff et al combined it with MADM reporter to sparsely label MGE-derived interneurons and found that a substantial proportion of the labeled neurons are ChCs [18]. Sultan et al combined it with a Cre-dependent TVAiLacZ reporter to facilitate infection of RCAS retroviruses into TVA-expressing MGE progenitors (Fig. 2B). Through extensive clonal analysis, they found that the late-born chandelier cells ChCs as the final interneuron output of MGE progenitors. Once again, the identification of ChCs in these studies relies on morphological features [24].

Although genetic fate mapping-based labeling has provided valuable insights into the developmental principles and connectivity patterns of ChCs, there is still a pressing need for improved genetic tools with higher labeling efficiency and specificity that can be readily applied in the postnatal brain. Recent single-cell and single-nucleus transcriptomic studies identified potential marker genes expressed in differentiated ChCs, leading to the generation of several mouse driver lines that do not rely on embryonic recombination: Unc5b-CreER, Pthlh-Flp, and Vipr2-Cre (Fig. 2C) [14, 15, 17, 25]. These driver liners are not entirely specific to ChCs when combined with reporter mouse lines. Unc5b-CreER labels a large number of endothelial cells in the blood vessels. Pthlh-Flp labels another major interneuron class expressing vasoactive intestinal peptide (VIP). Vipr2-Cre labels basket cells, Meis2-expressing GABAergic neurons in the white matter originating from the embryonic pallial–subpallial boundary, and many non-neuronal cells [15]. Among these three lines, Unc5b-CreER exhibits the highest labeling efficiency and enables highly specific labeling when combined with viral-reporters, or intersected with Nkx2.1-Flp to eliminate endothelial labeling (Fig. 2C) [17, 26]. It has been successfully used to label ChCs in the hippocampal CA1 region for calcium imaging and to map the distribution of ChCs throughout the brain [17, 27]. Intersecting Pthlh-Flp with Nkx2.1-Cre or Nkx2.1-CreER revealed a similar pattern to Unc5b-CreER intersected with Nkx2.1-Flp, albeit with slightly higher density in the retrosplenial cortex [17]. Vipr2-Cre has been employed to label ChCs in the neocortex and basolateral amygdala [15, 25, 28]. Combining it with viral reporters or intersecting with PV-Flp helps to enhance its labeling specificity, although not to the same extent as the other two lines [17]. These new labeling strategies, particularly those based on Unc5b-CreER, provide much-needed experimental access to this important cell type and hold great promise for advancing our understanding of ChC development and function in different brain regions in the near future.

## Specificity of the ChC-AIS Connection

As introduced in earlier sections, the most defining feature or ChCs is their remarkable connection specificity. ChCs exclusively target the AIS of local glutamatergic PNs, while avoiding other subcellular domains and GABAergic interneurons. This precise organization is believed to have significant functional implications. Understanding how this specificity is achieved and regulated has garnered much research attention. Before delving into recent progress on this front, we will first introduce the unique features of AISs (Fig. 3A).

### Structure and Function of AIS

The AIS is a highly specialized subcellular domain located at the proximal end of the axon. It is unmyelinated and has dense distribution of voltage-gated ion channels (Fig. 3A) [63–65]. Immunohistochemical staining and whole-cell membrane clamp recordings have revealed that the density of sodium channels at the AIS is 5-50 times higher than that found in the distal axons or dendrites, making it the most excitable compartment in a neuron and the site of action potential initiation [66, 67]. Therefore, the GABAergic synapses established by ChCs on the AIS are believed to play a critical role in regulating the excitability and output of the PNs. It is important to note that while ChCs exclusively target AISs, a substantial percentage of GABAergic synapses on the AIS are from non-ChCs [25, 51, 68]. Distinct from ChC-derived AIS connections, which often consist of several consecutive synapses within one axonal cartridge, most non-ChC-derived AIS connections contain only a single synapse. This highlights the importance of the ChC-AIS connections in regulating neuronal output. In addition to its role in generating action potential, the AIS also contributes to maintaining neuronal polarity [69, 70]. However, whether and how ChC-AIS connections may contribute to this aspect of AIS function remains unexplored.

Ankyrin G (AnkG), a member of the Ankyrin family cytoskeleton proteins, is the master organizer which acts as an anchor protein to recruit and organize other AIS-specific cytoskeletal and transmembrane proteins (Fig. 3A) [71–73]. AnkG exists in multiple isoforms, with the 270 kDa and 480 kDa isoforms being the major variants localized at the AIS and nodes of Ranvier [74, 75]. Knocking out AnkG disrupts the proper localization of other AIS proteins and perturbs neuronal polarity [71, 73, 76]. The 480 kDa isoform of AnkG is required for the maintenance of the AIS lattice structure [77]. Expressing the 480-kDa AnkG fully restores the AIS structure and the localization of known AIS binding proteins in AnkG-null neurons. However, expressing 270-kDa AnkG fails to fully restore the AIS structure, and only partially restores the localization of AIS binding proteins [74]. The Nav1 family voltage-sensitive ion channels are anchored to the AIS by AnkG through a cytosolic lipid loop containing an AnkG binding motif [78]. The clustering and anchoring of sodium channels at the AIS are facilitated by the AIS-enriched protease CK2, which increases the affinity of AnkG for sodium channels [79]. Different subtypes of sodium channels have distinct AIS distribution [67, 80, 81]. For instance, in cortical PNs, Nav1.2 with a higher activation threshold is enriched in the proximal AIS, while Nav1.6 with a lower activation threshold is enriched in the distal AIS [81]. Interestingly, ChCs mainly innervate the distal part of the AIS, which closely aligns with the distribution of Nav1.6 [82]. The properties of the AIS are not uniform and exhibit structural and molecular variability across different cell types [83]. Hu *et al.* comprehensively analyzed the AISs of projection-defined PN subtypes in superficial and deep layers in the mouse prelimbic cortex and found significant differences in their morphology, ion channel expression, action potential initiation, and axo-axonic synaptic inputs from ChCs [26]. Whether these different aspects may interact with one another needs further investigation. Nonetheless, AIS heterogeneity is an important aspect of neuronal diversity and contributes to the functional differences between different neuronal types.

### Principles of ChC-AIS Connections

Despite the highly specific nature, ChCs do not innervate every PN within their arborization. In the mouse somatosensory cortex, one study estimated the connection rate to be around 30%–50% based on the reconstruction of cartridges from individual ChCs and all AISs in their axonal fields [84]. A single ChC is capable of targeting hundreds of AISs, and a single AIS is usually innervated by multiple ChCs [25]. The number of axo-axonic synapses on each AIS is highly variable, ranging from zero to several dozen [25, 51]. This variability suggests a biased connection between ChCs and certain subpopulations of PNs.

Indeed, multiple studies have observed systematic differences in ChC connections between projection-based PN subtypes. An early study in the cat auditory and visual cortices showed higher synaptic densities on the AIS of callosal projection neurons (CPNs) than thalamic projection neurons (CThPNs), suggesting that ChCs may preferentially connect to CPNs over CThPNs (Fig. 3B) [85]. Another study showed that ChCs in the mouse piriform cortex may preferentially connect with centrifugal cells (FG) projecting to the main olfactory bulb (MOB) over non-centrifugal cells (nonFG) (Fig. 3C) [86]. However, these studies solely relied on anatomical analysis of synapses on the AIS to infer ChC connections and did not record the strength of functional connections.

A more recent study in the mouse prelimbic cortex by Lu *et al.* utilized paired whole-cell patch-clamp recordings to demonstrate the existence of preferential connections between ChCs and specific PN subtypes [19]. They labeled ChCs using late embryonic induction of Nkx2.1-CreER and two projection-defined PN subtypes using retrograde dye tracing: the BLA-projecting PNs (BLAPNs) and the CPNs projecting to the contralateral prelimbic cortex. The connection probability and synaptic strength indicated that ChCs preferentially innervate BLAPNs over CPNs (Fig. 3D) [19]. This discovery was further corroborated by a subsequent study by Hu *et al.* which quantified the number and distribution of axo-axonic synapses established by ChCs on the two PN subtypes [26]. They labelled ChCs using the intersection Unc5b-CreER and Nkx2.1-Flp (Fig. 2C). The fact that ChCs labeled by two different genetic methods and analyzed by two different techniques (recording and immunostaining) showed the same preferences strongly supports the notion that ChCs have target selectivity at the cell type level. However, the generalizability of this selectivity across other brain regions remains to be investigated.

Several studies have also observed diverse morphological ChCs subtypes, variable bouton density along cartridges, and significant laminal or regional differences in cartridge density [17, 47]. Whether and how these observations reflect or contribute to the subtype selectivity of ChC connections remains to be tested.

### Molecular Mechanisms Underlying the Specificity of ChC-AIS Connection

The establishment of the remarkably specific ChC-to-AIS connections in PNs involves the contribution of various molecular mediators. Several genes have been identified to participate in the establishment and maintenance of ChC-AIS connections, including Erbb4/Dock7, L1CAM, Fgf13, IgSF11, and Cntn-1 [57, 87–90]. While deficiencies in these genes have been shown to result in impairments in the maturation, density, or morphology of ChC boutons and cartridges, none of these deficiencies lead to mistargeting of ChC synapses to other cellular compartments or GABAergic interneurons, suggesting the major mediators responsible for synaptic specificity in ChC connections are yet to be discovered. Nonetheless, these studies have provided valuable insights and established methodologies that can be used to search for additional candidate molecules involved in ChC-AIS connections. The involvement of Erbb4 and Dock7 was identified through targeted manipulations of these genes [57], while the involvement of other molecules was discovered through screening-based approaches [88–90].

Tai *et al.* hypothesized that adhesion molecules enriched at the AIS might play critical roles in attracting ChCs to innervate them. To test this idea, they knocked down each of the known AIS-enriched adhesion molecules using RNAi vectors delivered into LayerII/III PNs of the somatosensory cortex through in-utero electroporation (IUE). Unexpectedly, none of the AIS-enriched adhesion molecules were found to impair the ChC-AIS innervation. Instead, L1CAM, a pan-axonal adhesion molecule, was identified as the key player in mediating the formation and maintenance of axo-axonic synapses between ChCs and AISs. Furthermore, they also discovered that L1CAM binds to AnkG, the master regulator of the AIS, which reduces the motility of L1CAM at the AIS and contributes to the stabilization of ChC-AIS innervation[87]. It remains unclear which protein serves as the binding partner for L1CAM at the presynaptic side. It is also worth noting that while L1CAM is also expressed in the axons of GABAergic interneurons, their AISs are not innerved by ChCs [25, 51, 91]. Therefore, L1CAM alone is not sufficient to explain why ChCs specifically target PN AISs.

The molecules screened by Tai *et al.* were only a small proportion of all AIS-located proteins. Two independent works used antibody-directed proximity biotinylation to identify cell surface proteins in close proximity to the AIS cell adhesion molecule neurofascin and discovered new candidates expressed at the AIS [90, 92]. Among them, the cell surface protein Cntn1 was found to be required for the axo-axonic innervation by ChCs. It remains to be investigated whether Cntn1 and L1CAM may cooperate with each other to regulate the assembly of ChC-AIS synapses and whether they share the same presynaptic receptors on ChCs.

On the presynaptic side, Favuzzi *et al.* analyzed transcriptome data from different interneuron cell types across development to identify genes with enriched expression in ChCs at early stages when synaptic contacts are established [88]. The rationale behind this analysis was that these genes might be involved in regulating the development of axo-axonic synapses. Among them, Fgf13, an intracellular protein with multiple functions including microtubule stabilization, emerged as a promising candidate. Knocking down Fgf13 resulted in axonal disorganization and decreased density of presynaptic inputs to the PN AIS. Similarly, another independent study using a similar approach of transcriptome analysis at early developmental stages in different interneuron subtypes identified the adhesion molecule IgSF11 as a candidate regulator of ChC-AIS connections [89]. This molecule is enriched in ChCs compared to VIP and somatostatin (SOM) interneuron types (data from PVBC are not available) and regulates the laminar selection of ChCs through homophilic trans-binding with IgSF11 expressed in PNs in the target lamina. Ectopic expression of IgSF11 in PNs of non-targeting lamina resulted in ChC axons innervating previously non-targeted PNs in the deeper cortical layer.

## Plasticity of the ChC-AIS Connection

### AIS Plasticity

The AIS is a highly plastic structure that dynamically adjusts neuronal excitability in response to changes in network activity. The morphology and molecular composition of the AIS have been extensively studied and shown to be remodeled to accommodate inputs and maintain network homeostasis. In cultured rat hippocampal neurons, chronic depolarization induced by the application of potassium chloride to the culture medium leads to the movement of the AIS towards the distal end of the axon, accompanied by a decrease in neuronal excitability [7]. Upon removal of potassium chloride, the AIS returns to its original position. In vivo studies have shown that deprivation of auditory input in avian brainstem auditory neurons results in the elongation of the AIS, accompanied by increased Nav1 channels at the AIS (Fig. 3E) [6]. Additionally, there is a shift in the major Kv channel subtype from Kv1.1 to Kv7.2, leading to an increase in neuronal excitability, which may compensate for the loss of sensory input [93]. It is worth noting that plastic changes in the AIS can occur rapidly, both in neuronal cultures and *in vivo* [8, 94]. For instance, exposure to an enriched environment can lead to a significant shortening of the AIS in Layer II/III PNs in the barrel cortex within a matter of hours (Fig. 3E) [8]. Interestingly, differences in AIS properties and plasticity have been observed between species. Hippocampal cultures derived from mice and Sprague-Dawley (SD rats) have shown variations in AP propagation speed, AnkG, and Nav1.2 distribution patterns. Furthermore, the AIS plasticity properties also differ, suggesting that species should be considered an important factor when studying AIS properties [95]

Most of the published work in this field has focused on characterizing the homeostatic changes that occur at the AIS in response to overall network activity changes. However, the specific impact of local changes in synaptic input on AIS plasticity is not known. Data from our laboratory suggest that chronic alterations in local axo-axonic synaptic activity through manipulations of ChCs alone are sufficient to induce homeostatic changes in the AIS. These plastic changes occurring at the AIS encompass both structural modifications and alterations in the expression level of Nav1 channels. This combination of changes can result in a shift in the action potential threshold and subsequently neuronal excitability. Interestingly, we have observed a correlation between the timing of homeostatic plasticity at the AIS and the recovery of behavior deficits[96]. This suggests that plasticity originating from a specific subcellular region, such as the AIS, in response to changes in a particular circuit input, can result in physiological consequences. Establishing a direct causal relationship between AIS plasticity and behavioral changes is still a challenging task. Further research and advancements in methodology are needed to fully elucidate the mechanisms underlying AIS plasticity and its connection to neuronal adaptation and the maintenance of proper brain function.

### ChC Plasticity During Development

During postnatal development, ChCs undergo significant apoptosis and a prolonged maturation process to establish their connections with PNs on the AISs [13, 29, 30, 87]. Several studies observed dynamic changes in the densities of ChC cartridges or synapses on the AISs at the population level during postnatal development [87]. The general trend is a gradual increase, reaching a peak followed by an extended plateau phase, and then a gradual decline. This trend has been observed in both rodent and primate brains [87, 97]. Consistent with the changes in cartridges, morphological analysis of ChCs labeled by genetic fate mapping also revealed the elaboration and pruning of ChC axons during postnatal development, accompanying increased bouton number per cartridge [25, 29]. Importantly, both apoptosis and morphological maturation can be modulated by sensory input and neural activity [29, 30].

Wang *et al.* showed that retinal and callosal activity modulates ChC apoptosis in the binocular region of the primary visual cortex (V1) during the second postnatal week. Reduced callosal projection activity blocks the apoptosis of ChCs and results in a contralateral eye-dominated V1 and deficient binocular vision, suggesting a central role of ChC in shaping experience-dependent tuning of sensory perception [30]. Pan-Vazquez *et al.* showed that chemogenetic activation of PNs increases in network activity resulted in a reduction of axo-axonic synapses during the synaptogenesis period. Yet, similar manipulations at later time points during early adulthood resulted in changes in the opposite direction (Fig. 3F) [29]. It is worth noting that alterations in axo-axonic connections may, in turn, induce AIS plasticity. Nevertheless, direct evidence is lacking to provide a clear understanding of the potential link between axo-axonic connection plasticity and AIS plasticity. The cellular and molecular mechanisms underlying these plastic changes remain to be uncovered. Gallo *et al.* showed that microglia participate in the proper formation and maintenance of synapses on AIS [98]. Microglia were well-known for their involvement in activity-dependent establishment and pruning of synapses, and thereby could also be an important mediator of the activity-induced ChC-AIS plasticity during development. Steinecke *et al.* showed that a neuromodulator, through the nicotinic receptor, plays an important role in the elaboration of ChC axon arbors during development [99]. The involvement of other neuromodulators and neurotransmitters in the developmental maturation and plasticity of ChCs awaits future exploration.

### ChC Function and Plasticity

What drives ChCs activity? Previous studies have indicated that both whisker stimulation-evoked and spontaneous IPSPs happen earlier than EPSPs in ChCs through in vivo whole-cell patches in the somatosensory cortex [61]. Additionally, ChCs exhibited small, long-latency initial EPSP followed by several delayed EPSPs. These features suggest that ChCs are not primarily involved in processing fast sensory inputs. Instead, they may monitor network excitability and only fire when there is excessive excitability in the network. ChCs can also respond to sensory input. Large-scale optical recordings have shown coordinated increases in ChC activity in the CA1 region of the hippocampus at the onset of locomotion and whisking episodes [27].

What are the functional consequences of ChC firing? Individual ChCs can innervate hundreds of PNs at their AISs, suggesting their potential involvement in coordinating network activity. In vivo, recording has demonstrated that ChCs in the CA3 region stopped firing during sharp-wave-ripples (SWR), which are associated with memory consolidation, but exhibit strong and rhythmic firing around the peak of theta oscillations [50, 55]. A subsequent study analyzed more ChCs using fast, targeted three-dimensional (3D) calcium imaging in the mouse CA3 region, confirming these earlier observations [27].

The suppression of ChC firing during SWR leads to a temporary reduction in GABA release onto the AISs of CA1 and CA3 PNs [50, 55]. This coordinated inhibition of cell assemblies is essential for their subsequent reactivation. CA3 ChCs are selectively inhibited by a subset of GABAergic medial septal neurons that exhibit strong firing around the trough of theta oscillation [55]. The rhythmic silencing of ChCs during theta oscillation leads to the disinhibition of PNs, thereby contributing to the generation of the theta dipole. Hence, ChCs in the CA3 region may serve as gatekeepers, regulating the firing of CA3 PNs during SWR and theta oscillation.

The correlated firing of ChCs is not limited to the hippocampus but has also been observed in the cortex. Using calcium imaging, Schneider-Mizell *et al.* observed highly synchronized activity among ChCs lasting for seconds in the primary visual cortex of awake-behaving animals [25]. Importantly, they found that the synchronized activation of ChCs correlated with pupil dilation, a sign of arousal [25]. Since PNs exhibit reduced spontaneous activity during arousal, this observation suggests that ChCs may suppress the spontaneous activity of PNs to enhance the signal-to-noise ratio during arousal.

In vivo, recording or functional imaging studies have provided valuable insights into the contribution of ChCs to specific behaviors or brain functions. The development of new genetic tools has enabled the specific modulation of ChC activity, leading to exciting discoveries about their functional roles. In a random foraging task, inhibiting ChCs in the CA1 region at specific locations resulted in place field remapping, indicating that ChCs are necessary for suppressing PN activity outside their respective place field [27]. In another study, blocking ChC synaptic transmission in the motor cortex results in perturbations in the precision and accuracy of directional movement control, indicating that ChCs are involved in fine-tuning the directional selectivity of premotor neurons [16]. Notably, increased heterogeneity in ChC-AIS connections was observed in this behavioral paradigm after motor learning, which may contribute to the formation and retrieval of memory. The plasticity at the presynaptic side, involving ChCs, may interact with AIS plasticity on the postsynaptic side to shape functional neural circuits. The commonality and significance of ChC-AIS plasticity in more complex functional settings are yet to be fully explored.

## ChC Plasticity in Brain Disorders

Pathological alterations in ChCs have been implicated in several neurological disorders, including epilepsy, schizophrenia, ASD, and AD [31, 32, 52, 54, 100]. These diseases are frequently associated with disturbances in overall network activity or higher-order brain functions. However, in human patients, especially in advanced stages of disease progression, distinguishing compensatory changes from disease-causing changes can be challenging. Therefore, it remains unclear whether the changes in ChCs accompany, facilitate, or counteract the pathological changes in these conditions. The newly developed genetic tools may enable functional manipulation of ChC in animal models to mimic and even reverse pathologic changes seen in human diseases, which will provide valuable insights into the etiology and open new avenues for the treatment of these diseases.

### Epilepsy

Inconsistencies and occasionally contradictory observations have been reported regarding the morphological changes of ChCs that happened in both patients and animal models of epilepsy [52, 100–102]. Variability in brain regions examined and the specific type or stage of epilepsy may contribute to these discrepancies. In the epileptogenic cortex of human patients with temporal lobe epilepsy (TLE), ChC loss is a common and prominent change [52]. However, subsequent research from the same laboratory reported that TLE patients with hippocampal sclerosis exhibited reorganized ChC cartridges [100]. These changes were mainly observed in the hippocampal formation and dentate gyrus, but not in the subiculum. In certain hippocampal segments, more complex and increased innervation by ChC terminals was observed, while in other regions showed a lack of labeling. This heterogeneity was evident not only between different patients but also within different subregions of the hippocampus within the same patient [100]. In a monkey model of cortical focal epilepsy, degeneration of ChC axonal terminals was observed [101]. In a recent study utilizing a mouse model, ChCs in the molecular layer of the dentate gyrus were labeled using PV-Cre, revealing reduced excitability of these ChCs following status epilepsy (SE) [33].

Do changes in ChCs promote or prevent epilepsy? Considering their GABAergic nature, ChCs are generally thought to prevent hyperexcitability, and increasing their output should have a protective effect. Importantly, in vivo recordings have shown that when the overall cortical excitability increases, ChCs are preferentially activated compared to other GABAergic neurons, suggesting a positive role in preventing hyperexcitability [61]. In this context, the reduction of ChCs in epileptic patients could be detrimental, as it may either initiate the ictal phase or propagate epileptiform discharges. Conversely, changes in the opposite direction, such as a higher number or more complex ChC cartridges, would provide more inhibition and work against epilepsy. The increased complexity of ChC terminals observed in hippocampal regions may be a compensatory effect aimed at counteracting hyperexcitability. However, studies have shown that altered chloride ion (Cl-) homeostasis caused by downregulated KCC2 and upregulated NKCC1 in the brain of TLE patients results in a depolarizing effect of GABA [103–105]. If this is the case, increased ChC cartridges might instead contribute to increased synchronization during epileptic activity. To further complicate matters, the shunting effect, which decreases the likelihood of firing regardless of the polarity of Cl- across the AIS membranes, must also be considered [106, 107]. Simulations using a multicompartmental granule cell model that included an AIS compartment revealed a compromised shunting effect of ChCs on the firing of granule cell action potentials after SE, supporting the idea that impaired ChC function in epilepsy could have detrimental effects. Future studies using animal models to specifically manipulate ChC activity will be crucial in determining the functional relevance of ChCs in epilepsy and resolving controversies [33].

### Schizophrenia

ChCs have long been proposed to participate in higher-order cognitive functions due to their restricted distribution in cortical structures in the mammalian brain. Consistent with this hypothesis, the reduction of ChCs and their synaptic terminals in the mPFC has been observed in postmortem brain tissue from patients with schizophrenia [32]. Studies in these patients have shown that the dorsolateral prefrontal cortex (dlPFC), a brain region involved in higher cognitive functions such as information processing, decision-making, and attention, showed a decrease in the expression of the GAT-1 transporter in ChC cartridges distributed in L2/3. Additionally, there is an increase in the expression of the GABA receptor α2 subunit at the AIS, which is most prominent in L2/3 [32, 108]. The negative correlation between presynaptic release transporters and postsynaptic receptors suggests a compensatory nature of these changes [109].

However, another study reported changes in the opposite direction. They found no changes in the number of boutons per cartridge or the expression level of GAD67 and VGAT in ChC boutons in L2 of the PFC in individuals with schizophrenia. However, they did observe an increased average density of VGAT+/CB+ cartridges in L2, not in L3-6 [110]. One possible explanation for this seemingly contradictory finding is that L2 matures later than the deep layers, and the increased density of VGAT+/CB+ cartridges may reflect abnormal axon pruning during late development. Despite the conflicting evidence, abnormalities in L2 ChCs have been observed in both studies, suggesting a potential close relationship between these abnormalities and schizophrenia.

### Autism Spectrum Disorder

Similar to schizophrenia, ASD is a neurodevelopmental disorder characterized by dysregulated higher-order cognitive functions. Abnormalities in ChCs have also been observed in ASD patients. In 2017, researchers found a significant reduction in PV neurons in specific regions of PFC in the brain tissue of ASD patients [111]. To determine which type of PV neurons were affected, ChCs or PVBCs, the researchers utilized PV staining along with VVA to distinguish the two of them. They found a decrease specifically in ChCs located in the granular layer of the BA9 and BA47 regions of the PFC [31].

In another experiment, the density of GAT-1+ cartridges of the ChCs was investigated in various regions of the PFC in ASD patients [53]. The results showed a decreased density of GAT-1+cartridges by approximately 40%–60% in different regions. Importantly, this reduction closely corresponded to the proportion of PV+ neuron reduction observed in the corresponding regions. These findings further support the notion that the decrease in the number of ChCs observed in the previous experiment is responsible for the observed changes, rather than a decrease in the protein expression level. Interestingly, animal models of ASD induced by adolescent isolated rearing also exhibited reduced ChC cartridges in the PFC [112], providing additional support for the functional role of ChCs in ASD. Based on the above research, it is important to note that further research is required to establish causal relationships between ChC abnormalities and ASD.

### Alzheimer’s Disease

AD is a neurodegenerative disease characterized by symptoms such as memory loss, difficulties with concentration, and problem-solving. While AD affects various cellular components with divergent outcomes, changes in the AIS have been observed in both animal models and patients [113–115]. However, regarding the axo-axonic synapses, the results are controversial, likely due to differences in animal models and the stage of disease progression. One study showed a 30%–35% reduction in cartridges in L2/3 of the human temporal cortex without changes in the number of PV-positive cells [54]. Similar findings have been reported in the APP/PS1 mouse model, in which a reduction in axo-axonic synapses forming on AISs that contact Aβ plaques was observed [116]. It is intriguing that in the APP mutant knock-in mouse model, which better mimics familiar AD, the total number of axo-axonic synapses remained unchanged, but their size was enlarged [117]. These observations, while not providing a clear understanding of how ChCs are involved in the etiology of AD, present an interesting direction for further exploration. Studying the plastic changes of ChCs may shed light on their potential contribution to memory loss and cognitive decline in AD.

## Summary

ChCs have garnered significant attention since their discovery more than 50 years ago due to their highly specificity innervation on the AISs of PNs. The functional investigation of ChCs had been impeded by the lack of experimental access for precise and efficient labeling and manipulation. Recent advancements in genetic tools have greatly facilitated the in-depth exploration of ChC development, connectivity, and activity, as well as their structural and functional plasticity. These plastic changes allow ChCs to effectively adapt to the changing environment during development or across various physiological and pathological conditions. Further research is necessary to fully elucidate the roles of ChC plasticity in diverse brain regions and under different circumstances, particularly to reconcile conflicting findings from previous studies. Investigating whether and how such changes may contribute to the maintenance of network homeostasis, the functional optimization of neural circuits, and the onset and progression of brain disorders may lead to exciting new findings.

## References

[1] Stuart G, Häusser M. Initiation and spread of sodium action potentials in cerebellar Purkinje cells. Neuron 1994, 13: 703–712. 7917300 10.1016/0896-6273(94)90037-x

[2] Colbert CM, Johnston D. Axonal action-potential initiation and Na^+^Channel densities in the soma and axon initial segment of subicular pyramidal neurons. J Neurosci 1996, 16: 6676–6686. 8824308 10.1523/JNEUROSCI.16-21-06676.1996 PMC6579266

[3] Clark BA, Monsivais P, Branco T, London M, Häusser M. The site of action potential initiation in cerebellar Purkinje neurons. Nat Neurosci 2005, 8: 137–139. 15665877 10.1038/nn1390

[4] Shu Y, Duque A, Yu Y, Haider B, McCormick DA. Properties of action-potential initiation in neocortical pyramidal cells: Evidence from whole cell axon recordings. J Neurophysiol 2007, 97: 746–760. 17093120 10.1152/jn.00922.2006

[5] Coombs JS, Curtis DR, Eccles JC. The generation of impulses in motoneurones. J Physiol 1957, 139: 232–249. 13492210 10.1113/jphysiol.1957.sp005888 PMC1358726

[6] Kuba H, Oichi Y, Ohmori H. Presynaptic activity regulates Na(+) channel distribution at the axon initial segment. Nature 2010, 465: 1075–1078. 20543825 10.1038/nature09087

[7] Grubb MS, Burrone J. Activity-dependent relocation of the axon initial segment fine-tunes neuronal excitability. Nature 2010, 465: 1070–1074. 20543823 10.1038/nature09160 PMC3196626

[8] Jamann N, Dannehl D, Lehmann N, Wagener R, Thielemann C, Schultz C, *et al*. Sensory input drives rapid homeostatic scaling of the axon initial segment in mouse barrel cortex. Nat Commun 2021, 12: 23. 33397944 10.1038/s41467-020-20232-x PMC7782484

[9] Somogyi P, Freund TF, Hodgson AJ, Somogyi J, Beroukas D, Chubb IW. Identified axo-axonic cells are immunoreactive for GABA in the hippocampus visual cortex of the cat. Brain Res 1985, 332: 143–149. 3995258 10.1016/0006-8993(85)90397-x

[10] McDonald AJ, Culberson JL. Neurons of the basolateral amygdala: A Golgi study in the opossum (*Didelphis virginiana*). Am J Anat 1981, 162: 327–342. 7325125 10.1002/aja.1001620404

[11] Jones EG. Varieties and distribution of non-pyramidal cells in the somatic sensory cortex of the squirrel monkey. J Comp Neurol 1975, 160: 205–267. 803518 10.1002/cne.901600204

[12] Szentágothai J, Arbib MA. Conceptual models of neural organization. Neurosci Res Program Bull 1974, 12: 305–510. 4437759

[13] Taniguchi H, Lu J, Huang ZJ. The spatial and temporal origin of chandelier cells in mouse neocortex. Science 2013, 339: 70–74. 23180771 10.1126/science.1227622 PMC4017638

[14] Paul A, Crow M, Raudales R, He M, Gillis J, Huang ZJ. Transcriptional architecture of synaptic communication delineates GABAergic neuron identity. Cell 2017, 171: 522–539.e20. 28942923 10.1016/j.cell.2017.08.032 PMC5772785

[15] Tasic B, Yao Z, Graybuck LT, Smith KA, Nguyen TN, Bertagnolli D, *et al*. Shared and distinct transcriptomic cell types across neocortical areas. Nature 2018, 563: 72–78. 30382198 10.1038/s41586-018-0654-5 PMC6456269

[16] Jung K, Chang M, Steinecke A, Burke B, Choi Y, Oisi Y, *et al*. An adaptive behavioral control motif mediated by cortical axo-axonic inhibition. Nat Neurosci 2023, 26: 1379–1393. 37474640 10.1038/s41593-023-01380-x PMC10400431

[17] Raudales R, Kim G, Kelly SM, Hatfield J, Guan W, Zhao S, *et al*. Specific and comprehensive genetic targeting reveals brain-wide distribution and synaptic input patterns of GABAergic axo-axonic interneurons. bioRxiv 2024: 2023.11.07.566059. 10.7554/eLife.93481 PMC11251723 39012795

[18] Woodruff A, Xu Q, Anderson SA, Yuste R. Depolarizing effect of neocortical chandelier neurons. Front Neural Circuits 2009, 3: 15. 19876404 10.3389/neuro.04.015.2009 PMC2769545

[19] Lu J, Tucciarone J, Padilla-Coreano N, He M, Gordon JA, Huang ZJ. Selective inhibitory control of pyramidal neuron ensembles and cortical subnetworks by chandelier cells. Nat Neurosci 2017, 20: 1377–1383. 28825718 10.1038/nn.4624 PMC5614838

[20] He M, Huang ZJ. Genetic approaches to access cell types in mammalian nervous systems. Curr Opin Neurobiol 2018, 50: 109–118. 29471215 10.1016/j.conb.2018.02.003 PMC5984678

[21] Gong L, Liu X, Wu J, He M. Emerging strategies for the genetic dissection of gene functions, cell types, and neural circuits in the mammalian brain. Mol Psychiatry 2022, 27: 422–435. 34561609 10.1038/s41380-021-01292-x

[22] He M, Tucciarone J, Lee S, Nigro M, Kim Y, Levine J, *et al*. Strategies and tools for combinatorial targeting of GABAergic neurons in mouse cerebral cortex. Neuron 2016, 91: 1228–1243. 27618674 10.1016/j.neuron.2016.08.021 PMC5223593

[23] Yang JM, Shen CJ, Chen XJ, Kong Y, Liu YS, Li XW, *et al*. erbb4 deficits in chandelier cells of the medial prefrontal cortex confer cognitive dysfunctions: Implications for schizophrenia. Cereb Cortex 2019, 29: 4334–4346. 30590426 10.1093/cercor/bhy316

[24] Sultan KT, Liu WA, Li ZL, Shen Z, Li Z, Zhang XJ, *et al*. Progressive divisions of multipotent neural progenitors generate late-born chandelier cells in the neocortex. Nat Commun 2018, 9: 4595. 30389944 10.1038/s41467-018-07055-7 PMC6214958

[25] Schneider-Mizell CM, Bodor AL, Collman F, Brittain D, Bleckert A, Dorkenwald S, *et al*. Structure and function of axo-axonic inhibition. Elife 2021, 10: e73783. 34851292 10.7554/eLife.73783 PMC8758143

[26] Hu A, Zhao R, Ren B, Li Y, Lu J, Tai Y. Projection-specific heterogeneity of the axon initial segment of pyramidal neurons in the prelimbic cortex. Neurosci Bull 2023, 39: 1050–1068. 36849716 10.1007/s12264-023-01038-5 PMC10313623

[27] Dudok B, Szoboszlay M, Paul A, Klein PM, Liao Z, Hwaun E, *et al*. Recruitment and inhibitory action of hippocampal axo-axonic cells during behavior. Neuron 2021, 109: 3838–3850.e8. 34648750 10.1016/j.neuron.2021.09.033 PMC8639676

[28] Nakashima M, Ikegaya Y, Morikawa S. Genetic labeling of axo-axonic cells in the basolateral amygdala. Neurosci Res 2022, 178: 33–40. 35189175 10.1016/j.neures.2022.02.002

[29] Pan-Vazquez A, Wefelmeyer W, Gonzalez Sabater V, Neves G, Burrone J. Activity-dependent plasticity of axo-axonic synapses at the axon initial segment. Neuron 2020, 106: 265–276.e6. 32109363 10.1016/j.neuron.2020.01.037 PMC7181187

[30] Wang BS, Bernardez Sarria MS, An X, He M, Alam NM, Prusky GT, *et al*. Retinal and callosal activity-dependent chandelier cell elimination shapes binocularity in primary visual cortex. Neuron 2021, 109: 502–515.e7. 33290732 10.1016/j.neuron.2020.11.004 PMC7943176

[31] Ariza J, Rogers H, Hashemi E, Noctor SC, Martínez-Cerdeño V. The number of chandelier and basket cells are differentially decreased in prefrontal cortex in autism. Cereb Cortex 2018, 28: 411–420. 28122807 10.1093/cercor/bhw349 PMC6676950

[32] Woo TU, Whitehead RE, Melchitzky DS, Lewis DA. A subclass of prefrontal γ-aminobutyric acid axon terminals are selectively altered in schizophrenia. Proc Natl Acad Sci U S A 1998, 95: 5341–5346. 9560277 10.1073/pnas.95.9.5341 PMC20262

[33] Proddutur A, Nguyen S, Yeh CW, Gupta A, Santhakumar V. Reclusive chandeliers: Functional isolation of dentate axo-axonic cells after experimental status epilepticus. Prog Neurobiol 2023, 231: 102542. 37898313 10.1016/j.pneurobio.2023.102542 PMC10842856

[34] Ramón y Cajal S, Pasik P, Pasik T. Texture of the nervous system of man and the vertebrates. Springer, Wien 1999.

[35] Szentágothai J. The ‘module-concept’ in cerebral cortex architecture. Brain Res 1975, 95: 475–496. 808252 10.1016/0006-8993(75)90122-5

[36] Somogyi P. A specific ‘axo-axonal’ interneuron in the visual cortex of the rat. Brain Res 1977, 136: 345–350. 922488 10.1016/0006-8993(77)90808-3

[37] Gulyás AI, Miles R, Hájos N, Freund TF. Precision and variability in postsynaptic target selection of inhibitory cells in the hippocampal CA3 region. Eur J Neurosci 1993, 5: 1729–1751. 8124523 10.1111/j.1460-9568.1993.tb00240.x

[38] Fairén A, Valverde F. A specialized type of neuron in the visual cortex of cat: A Golgi and electron microscope study of chandelier cells. J Comp Neurol 1980, 194: 761–779. 7204642 10.1002/cne.901940405

[39] Müller-Paschinger IB, Tömböl T, Petsche H. Chandelier neurons within the rabbits' cerebral cortex. A Golgi study. Anat Embryol 1983, 166: 149–154. 10.1007/BF00317950 6837931

[40] Chiu CS, Jensen K, Sokolova I, Wang D, Li M, Deshpande P, *et al*. Number, density, and surface/cytoplasmic distribution of GABA transporters at presynaptic structures of knock-in mice carrying GABA transporter subtype 1-green fluorescent protein fusions. J Neurosci 2002, 22: 10251–10266. 12451126 10.1523/JNEUROSCI.22-23-10251.2002 PMC6758747

[41] Krimer LS, Goldman-Rakic PS. Prefrontal microcircuits: Membrane properties and excitatory input of local, medium, and wide arbor interneurons. J Neurosci 2001, 21: 3788–3796. 11356867 10.1523/JNEUROSCI.21-11-03788.2001 PMC6762691

[42] Somogyi P, Freund TF, Cowey A. The axo-axonic interneuron in the cerebral cortex of the rat, cat and monkey. Neuroscience 1982, 7: 2577–2607. 7155343 10.1016/0306-4522(82)90086-0

[43] Somogyi P, Nunzi MG, Gorio A, Smith AD. A new type of specific interneuron in the monkey hippocampus forming synapses exclusively with the axon initial segments of pyramidal cells. Brain Res 1983, 259: 137–142. 6824927 10.1016/0006-8993(83)91076-4

[44] Kisvárday ZF, Adams CB, Smith AD. Synaptic connections of axo-axonic (chandelier) cells in human epileptic temporal cortex. Neuroscience 1986, 19: 1179–1186. 3029627 10.1016/0306-4522(86)90131-4

[45] Li XG, Somogyi P, Tepper JM, Buzsáki G. Axonal and dendritic arborization of an intracellularly labeled chandelier cell in the CA1 region of rat hippocampus. Exp Brain Res 1992, 90: 519–525. 1385200 10.1007/BF00230934

[46] Bienvenu TCM, Busti D, Magill PJ, Ferraguti F, Capogna M. Cell-type-specific recruitment of amygdala interneurons to hippocampal theta rhythm and noxious stimuli *in vivo*. Neuron 2012, 74: 1059–1074. 22726836 10.1016/j.neuron.2012.04.022 PMC3391683

[47] Wang X, Tucciarone J, Jiang S, Yin F, Wang BS, Wang D, *et al*. Genetic single neuron anatomy reveals fine granularity of cortical axo-axonic cells. Cell Rep 2019, 26: 3145–3159.e5. 30865900 10.1016/j.celrep.2019.02.040 PMC7863572

[48] Chen A, Sun Y, Lei Y, Li C, Liao S, Meng J, *et al*. Single-cell spatial transcriptome reveals cell-type organization in the macaque cortex. Cell 2023, 186: 3726–3743.e24. 37442136 10.1016/j.cell.2023.06.009

[49] Miyamae T, Chen K, Lewis DA, Gonzalez-Burgos G. Distinct physiological maturation of parvalbumin-positive neuron subtypes in mouse prefrontal cortex. J Neurosci 2017, 37: 4883–4902. 28408413 10.1523/JNEUROSCI.3325-16.2017 PMC5426180

[50] Klausberger T, Magill PJ, Márton LF, Cobden PM, Buzsáki G, *et al*. Brain-state- and cell-type-specific firing of hippocampal interneurons *in vivo*. Nature 2003, 421: 844–848. 12594513 10.1038/nature01374

[51] Gour A, Boergens KM, Heike N, Hua Y, Laserstein P, Song K, *et al*. Postnatal connectomic development of inhibition in mouse barrel cortex. Science 2021, 371: eabb4534. 33273061 10.1126/science.abb4534

[52] Marco P, Sola RG, Pulido P, Alijarde MT, Sánchez A, *et al*. Inhibitory neurons in the human epileptogenic temporal neocortex. An immunocytochemical study. Brain 1996, 119 ( Pt 4): 1327–1347. 8813295 10.1093/brain/119.4.1327

[53] Amina S, Falcone C, Hong T, Wolf-Ochoa MW, Vakilzadeh G, Allen E, *et al*. Chandelier cartridge density is reduced in the prefrontal cortex in autism. Cereb Cortex 2021, 31: 2944–2951. 33527113 10.1093/cercor/bhaa402 PMC8107784

[54] Fonseca M, Soriano E, Ferrer I, Martinez A, Tuñon T. Chandelier cell axons identified by parvalbumin-immunoreactivity in the normal human temporal cortex and in Alzheimer’s disease. Neuroscience 1993, 55: 1107–1116. 8232900 10.1016/0306-4522(93)90324-9

[55] Viney TJ, Lasztoczi B, Katona L, Crump MG, Tukker JJ, Klausberger T, *et al*. Network state-dependent inhibition of identified hippocampal CA3 axo-axonic cells *in vivo*. Nat Neurosci 2013, 16: 1802–1811. 24141313 10.1038/nn.3550 PMC4471148

[56] Inan M, Welagen J, Anderson SA. Spatial and temporal bias in the mitotic origins of somatostatin- and parvalbumin-expressing interneuron subgroups and the chandelier subtype in the medial ganglionic eminence. Cereb Cortex 2012, 22: 820–827. 21693785 10.1093/cercor/bhr148 PMC3450921

[57] Tai Y, Janas JA, Wang CL, Van Aelst L. Regulation of chandelier cell cartridge and bouton development via DOCK7-mediated ErbB4 activation. Cell Rep 2014, 6: 254–263. 24440718 10.1016/j.celrep.2013.12.034 PMC3920736

[58] Fazzari P, Paternain AV, Valiente M, Pla R, Luján R, Lloyd K, *et al*. Control of cortical GABA circuitry development by Nrg1 and ErbB4 signalling. Nature 2010, 464: 1376–1380. 20393464 10.1038/nature08928

[59] Han ZS, Buhl EH, Lörinczi Z, Somogyi P. A high degree of spatial selectivity in the axonal and dendritic domains of physiologically identified local-circuit neurons in the dentate gyrus of the rat hippocampus. Eur J Neurosci 1993, 5: 395–410. 8261117 10.1111/j.1460-9568.1993.tb00507.x

[60] Buhl EH, Han ZS, Lörinczi Z, Stezhka VV, Karnup SV, Somogyi P. Physiological properties of anatomically identified axo-axonic cells in the rat hippocampus. J Neurophysiol 1994, 71: 1289–1307. 8035215 10.1152/jn.1994.71.4.1289

[61] Zhu Y, Stornetta RL, Zhu JJ. Chandelier cells control excessive cortical excitation: Characteristics of whisker-evoked synaptic responses of layer 2/3 nonpyramidal and pyramidal neurons. J Neurosci 2004, 24: 5101–5108. 15175379 10.1523/JNEUROSCI.0544-04.2004 PMC6729194

[62] Zhang Q, Liu X, Gong L, He M. Combinatorial genetic strategies for dissecting cell lineages, cell types, and gene function in the mouse brain. Dev Growth Differ 2023, 65: 546–553. 37963088 10.1111/dgd.12902

[63] Catterall WA. Localization of sodium channels in cultured neural cells. J Neurosci 1981, 1: 777–783. 6286901 10.1523/JNEUROSCI.01-07-00777.1981 PMC6564194

[64] Nusser Z. Variability in the subcellular distribution of ion channels increases neuronal diversity. Trends Neurosci 2009, 32: 267–274. 19299025 10.1016/j.tins.2009.01.003

[65] Bean BP. The action potential in mammalian central neurons. Nat Rev Neurosci 2007, 8: 451–465. 17514198 10.1038/nrn2148

[66] Kole MHP, Stuart GJ. Is action potential threshold lowest in the axon? Nat Neurosci 2008, 11: 1253–1255. 18836442 10.1038/nn.2203

[67] Lorincz A, Nusser Z. Molecular identity of dendritic voltage-gated sodium channels. Science 2010, 328: 906–909. 20466935 10.1126/science.1187958 PMC3546315

[68] Gonchar Y, Turney S, Price JL, Burkhalter A. Axo-axonic synapses formed by somatostatin-expressing GABAergic neurons in rat and monkey visual cortex. J Comp Neurol 2002, 443: 1–14. 11793343 10.1002/cne.1425

[69] Eichel K, Shen K. The function of the axon initial segment in neuronal polarity. Dev Biol 2022, 489: 47–54. 35640681 10.1016/j.ydbio.2022.05.016

[70] Szu-Yu Ho T, Rasband MN. Maintenance of neuronal polarity. Dev Neurobiol 2011, 71: 474–482. 21557501 10.1002/dneu.20843 PMC3117984

[71] Hedstrom KL, Ogawa Y, Rasband MN. AnkyrinG is required for maintenance of the axon initial segment and neuronal polarity. J Cell Biol 2008, 183: 635–640. 19001126 10.1083/jcb.200806112 PMC2582894

[72] Jenkins SM, Bennett V. Ankyrin-G coordinates assembly of the spectrin-based membrane skeleton, voltage-gated sodium channels, and L1 CAMs at Purkinje neuron initial segments. J Cell Biol 2001, 155: 739–746. 11724816 10.1083/jcb.200109026 PMC2150881

[73] Sobotzik JM, Sie JM, Politi C, Del Turco D, Bennett V, Deller T, *et al*. AnkyrinG is required to maintain axo-dendritic polarity *in vivo*. Proc Natl Acad Sci U S A 2009, 106: 17564–17569. 19805144 10.1073/pnas.0909267106 PMC2765162

[74] Jenkins PM, Kim N, Jones SL, Tseng WC, Svitkina TM, Yin HH, *et al*. Giant ankyrin-G: A critical innovation in vertebrate evolution of fast and integrated neuronal signaling. Proc Natl Acad Sci U S A 2015, 112: 957–964. 25552556 10.1073/pnas.1416544112 PMC4313853

[75] Zhang X, Bennett V. Restriction of 480/270-kD ankyrin G to axon proximal segments requires multiple ankyrin G-specific domains. J Cell Biol 1998, 142: 1571–1581. 9744885 10.1083/jcb.142.6.1571 PMC2141775

[76] Yang R, Walder-Christensen KK, Lalani S, Yan H, García-Prieto ID, Álvarez S, *et al*. Neurodevelopmental mutation of giant ankyrin-G disrupts a core mechanism for axon initial segment assembly. Proc Natl Acad Sci U S A 2019, 116: 19717–19726. 31451636 10.1073/pnas.1909989116 PMC6765234

[77] Wang Y, Guan M, Wang H, Li Y, Zhanghao K, Xi P, *et al*. The largest isoform of Ankyrin-G is required for lattice structure of the axon initial segment. Biochem Biophys Res Commun 2021, 578: 28–34. 34534742 10.1016/j.bbrc.2021.09.017

[78] Garrido JJ, Giraud P, Carlier E, Fernandes F, Moussif A, Fache MP, *et al*. A targeting motif involved in sodium channel clustering at the axonal initial segment. Science 2003, 300: 2091–2094. 12829783 10.1126/science.1085167

[79] Bréchet A, Fache MP, Brachet A, Ferracci G, Baude A, Irondelle M, *et al*. Protein kinase CK2 contributes to the organization of sodium channels in axonal membranes by regulating their interactions with ankyrin G. J Cell Biol 2008, 183: 1101–1114. 19064667 10.1083/jcb.200805169 PMC2600743

[80] Van Wart A, Trimmer JS, Matthews G. Polarized distribution of ion channels within microdomains of the axon initial segment. J Comp Neurol 2007, 500: 339–352. 17111377 10.1002/cne.21173

[81] Hu W, Tian C, Li T, Yang M, Hou H, Shu Y. Distinct contributions of Na(v)_1.6_ and Na(v)_1.2_ in action potential initiation and backpropagation. Nat Neurosci 2009, 12: 996–1002. 19633666 10.1038/nn.2359

[82] Tian C, Wang K, Ke W, Guo H, Shu Y. Molecular identity of axonal sodium channels in human cortical pyramidal cells. Front Cell Neurosci 2014, 8: 297. 25294986 10.3389/fncel.2014.00297 PMC4172021

[83] Höfflin F, Jack A, Riedel C, Mack-Bucher J, Roos J, Corcelli C, *et al*. Heterogeneity of the axon initial segment in interneurons and pyramidal cells of rodent visual cortex. Front Cell Neurosci 2017, 11: 332. 29170630 10.3389/fncel.2017.00332 PMC5684645

[84] Inan M, Blázquez-Llorca L, Merchán-Pérez A, Anderson SA, DeFelipe J, Yuste R. Dense and overlapping innervation of pyramidal neurons by chandelier cells. J Neurosci 2013, 33: 1907–1914. 23365230 10.1523/JNEUROSCI.4049-12.2013 PMC3711719

[85] Fariñas I, DeFelipe J. Patterns of synaptic input on corticocortical and corticothalamic cells in the cat visual cortex. II. The axon initial segment. J Comp Neurol 1991, 304: 70–77. 2016413 10.1002/cne.903040106

[86] Wang X, Sun QQ. Characterization of axo-axonic synapses in the piriform cortex of mus musculus. J Comp Neurol 2012, 520: 832–847. 22020781 10.1002/cne.22792 PMC3903392

[87] Tai Y, Gallo NB, Wang M, Yu JR, Van Aelst L. Axo-axonic innervation of neocortical pyramidal neurons by GABAergic chandelier cells requires AnkyrinG-associated L1CAM. Neuron 2019, 102: 358–372.e9. 30846310 10.1016/j.neuron.2019.02.009 PMC6525570

[88] Favuzzi E, Deogracias R, Marques-Smith A, Maeso P, Jezequel J, Exposito-Alonso D, *et al*. Distinct molecular programs regulate synapse specificity in cortical inhibitory circuits. Science 2019, 363: 413–417. 30679375 10.1126/science.aau8977

[89] Hayano Y, Ishino Y, Hyun JH, Orozco CG, Steinecke A, Potts E, *et al*. IgSF11 homophilic adhesion proteins promote layer-specific synaptic assembly of the cortical interneuron subtype. Sci Adv 2021, 7: eabf1600. 34261648 10.1126/sciadv.abf1600 PMC8279514

[90] Ogawa Y, Lim BC, George S, Oses-Prieto JA, Rasband JM, Eshed-Eisenbach Y, *et al*. Antibody-directed extracellular proximity biotinylation reveals that Contactin-1 regulates axo-axonic innervation of axon initial segments. Nat Commun 2023, 14: 6797. 37884508 10.1038/s41467-023-42273-8 PMC10603070

[91] Guan H, Maness PF. Perisomatic GABAergic innervation in prefrontal cortex is regulated by ankyrin interaction with the L1 cell adhesion molecule. Cereb Cortex 2010, 20: 2684–2693. 20156840 10.1093/cercor/bhq016 PMC2981022

[92] Zhang W, Fu Y, Peng L, Ogawa Y, Ding X, Rasband A, *et al*. Immunoproximity biotinylation reveals the axon initial segment proteome. Nat Commun 2023, 14: 8201. 38081810 10.1038/s41467-023-44015-2 PMC10713531

[93] Kuba H, Yamada R, Ishiguro G, Adachi R. Redistribution of Kv1 and Kv7 enhances neuronal excitability during structural axon initial segment plasticity. Nat Commun 2015, 6: 8815. 26581625 10.1038/ncomms9815 PMC4673506

[94] Evans MD, Dumitrescu AS, Kruijssen DLH, Taylor SE, Grubb MS. Rapid modulation of axon initial segment length influences repetitive spike firing. Cell Rep 2015, 13: 1233–1245. 26526995 10.1016/j.celrep.2015.09.066 PMC4646840

[95] Chen ZY, Peng L, Zhao M, Li Y, Takahiko M, Tao L, *et al*. Differences in action potential propagation speed and axon initial segment plasticity between neurons from Sprague-Dawley rats and C57BL/6 mice. Zool Res 2022, 43: 615–633. 35758537 10.24272/j.issn.2095-8137.2022.121 PMC9336440

[96] Zhao R, Ren B, Xiao Y, Tian J, Zou Y, Wei J, *et al*. Axo-axonic synaptic input drives homeostatic plasticity by tuning the axon initial segment structurally and functionally. bioRxiv 2024: 2024.04.11.589005. 10.1126/sciadv.adk4331 PMC11296346 39093969

[97] Cruz DA, Weaver CL, Lovallo EM, Melchitzky DS, Lewis DA. Selective alterations in postsynaptic markers of chandelier cell inputs to cortical pyramidal neurons in subjects with schizophrenia. Neuropsychopharmacology 2009, 34: 2112–2124. 19322171 10.1038/npp.2009.36 PMC2721024

[98] Gallo NB, Berisha A, Van Aelst L. Microglia regulate chandelier cell axo-axonic synaptogenesis. Proc Natl Acad Sci U S A 2022, 119: e2114476119. 35263225 10.1073/pnas.2114476119 PMC8931231

[99] Steinecke A, Bolton MM, Taniguchi H. Neuromodulatory control of inhibitory network arborization in the developing postnatal neocortex. Sci Adv 2022, 8: eabe7192. 35263136 10.1126/sciadv.abe7192 PMC8906727

[100] Arellano JI, Muñoz A, Ballesteros-Yáñez I, Sola RG, DeFelipe J. Histopathology and reorganization of chandelier cells in the human epileptic sclerotic hippocampus. Brain 2004, 127: 45–64. 14534159 10.1093/brain/awh004

[101] Ribak CE. Axon terminals of GABAergic chandelier cells are lost at epileptic foci. Brain Res 1985, 326: 251–260. 2982461 10.1016/0006-8993(85)90034-4

[102] Ferrer I, Pineda M, Tallada M, Oliver B, Russi A, Oller L, *et al*. Abnormal local-circuit neurons in epilepsia partialis continua associated with focal cortical dysplasia. Acta Neuropathol 1992, 83: 647–652. 1636380 10.1007/BF00299415

[103] Cohen I, Navarro V, Clemenceau S, Baulac M, Miles R. On the origin of interictal activity in human temporal lobe epilepsy *in vitro*. Science 2002, 298: 1418–1421. 12434059 10.1126/science.1076510

[104] Huberfeld G, Wittner L, Clemenceau S, Baulac M, Kai K, Miles R, *et al*. Perturbed chloride homeostasis and GABAergic signaling in human temporal lobe epilepsy. J Neurosci 2007, 27: 9866–9873. 17855601 10.1523/JNEUROSCI.2761-07.2007 PMC6672644

[105] Palma E, Amici M, Sobrero F, Spinelli G, Di Angelantonio S, Ragozzino D, *et al*. Anomalous levels of Cl- transporters in the hippocampal subiculum from temporal lobe epilepsy patients make GABA excitatory. Proc Natl Acad Sci U S A 2006, 103: 8465–8468. 16709666 10.1073/pnas.0602979103 PMC1482515

[106] Shang Z, Huang J, Liu N, Zhang X. Bi-directional control of synaptic input summation and spike generation by GABAergic inputs at the axon initial segment. Neurosci Bull 2023, 39: 1–13. 35639277 10.1007/s12264-022-00887-w PMC9849666

[107] Lipkin AM, Bender KJ. Axon initial segment GABA inhibits action potential generation throughout periadolescent development. J Neurosci 2023, 43: 6357–6368. 37596053 10.1523/JNEUROSCI.0605-23.2023 PMC10500977

[108] Volk DW, Pierri JN, Fritschy JM, Auh S, Sampson AR, Lewis DA. Reciprocal alterations in pre- and postsynaptic inhibitory markers at chandelier cell inputs to pyramidal neurons in schizophrenia. Cereb Cortex 2002, 12: 1063–1070. 12217970 10.1093/cercor/12.10.1063

[109] Lewis DA, Hashimoto T, Volk DW. Cortical inhibitory neurons and schizophrenia. Nat Rev Neurosci 2005, 6: 312–324. 15803162 10.1038/nrn1648

[110] Rocco BR, DeDionisio AM, Lewis DA, Fish KN. Alterations in a unique class of cortical chandelier cell axon cartridges in schizophrenia. Biol Psychiatry 2017, 82: 40–48. 27884423 10.1016/j.biopsych.2016.09.018 PMC5374057

[111] Hashemi E, Ariza J, Rogers H, Noctor SC, Martínez-Cerdeño V. The number of parvalbumin-expressing interneurons is decreased in the prefrontal cortex in autism. Cereb Cortex 2017, 27: 1931–1943. 26922658 10.1093/cercor/bhw021 PMC6074948

[112] Bloomfield C, French SJ, Jones DNC, Reavill C, Southam E, Cilia J, *et al*. Chandelier cartridges in the prefrontal cortex are reduced in isolation reared rats. Synapse 2008, 62: 628–631. 18512213 10.1002/syn.20521

[113] Marin MA, Ziburkus J, Jankowsky J, Rasband MN. Amyloid-β plaques disrupt axon initial segments. Exp Neurol 2016, 281: 93–98. 27109181 10.1016/j.expneurol.2016.04.018 PMC4877279

[114] Antón-Fernández A, León-Espinosa G, DeFelipe J, Muñoz A. Pyramidal cell axon initial segment in Alzheimer´s disease. Sci Rep 2022, 12: 8722. 35610289 10.1038/s41598-022-12700-9 PMC9130508

[115] Hatch RJ, Wei Y, Xia D, Götz J. Hyperphosphorylated tau causes reduced hippocampal CA1 excitability by relocating the axon initial segment. Acta Neuropathol 2017, 133: 717–730. 28091722 10.1007/s00401-017-1674-1 PMC5389999

[116] León-Espinosa G, DeFelipe J, Muñoz A. Effects of amyloid-β plaque proximity on the axon initial segment of pyramidal cells. J Alzheimers Dis 2012, 29: 841–852. 22337828 10.3233/JAD-2012-112036

[117] Sos KE, Mayer MI, Takács VT, Major A, Bardóczi Z, Beres BM, *et al*. Amyloid β induces interneuron-specific changes in the hippocampus of APPNL-F mice. PLoS One 2020, 15: e0233700. 32469963 10.1371/journal.pone.0233700 PMC7259556

